# Symptomatic syndrome of inappropriate anti-diuretic hormone as a rare early presentation of primary thymic carcinoma: a case report

**DOI:** 10.1093/jscr/rjae025

**Published:** 2024-02-09

**Authors:** Muhammad Juffri Samsuddin, Siti Sara Yaacob, Adli Azam Bin Mohammad Razi

**Affiliations:** Department of Cardiovascular & Thoracic Surgery, Faculty of Medicine, Universiti Teknologi MARA, Sungai Buloh Campus, 47000 Selangor, Malaysia; Department of Public Health Medicine, Faculty of Medicine, Universiti Teknologi MARA, Sungai Buloh Campus, Selangor Branch, Jalan Hospital, Selangor 47000, Malaysia; Department of Cardiovascular & Thoracic Surgery, Faculty of Medicine, Universiti Teknologi MARA, Sungai Buloh Campus, 47000 Selangor, Malaysia

**Keywords:** cardiothoracic surgery, syndrome of inappropriate anti-diuretic hormone (SIADH), thymic carcinoma, neoplasm, hyponatremia, mediastinal mass

## Abstract

Syndrome of inappropriate anti-diuretic hormone (SIADH) can be presented as a paraneoplastic syndrome in primary malignancies involving the lung and brain. However, the development of SIADH in primary thymic carcinoma is poorly documented. We report a case of an elderly, with an initial presentation of symptomatic persistent hyponatremia as a paraneoplastic syndrome of SIADH with an incidental finding of anterior mediastinal mass confirmed on imaging. Further investigations are consistent with the diagnosis of poorly differentiated locally advanced thymic carcinoma with lung infiltration (T3N1Mx). The patient underwent an En-bloc total thymectomy and subsequently completed adjuvant chemotherapy and further follow-up showed a complete resolution of hyponatraemic SIADH. In conclusion, SIADH may be presented as a paraneoplastic syndrome in primary thymic carcinoma and early detection of thymic malignancy is paramount to ensure early diagnosis and prognostication.

## Introduction

Syndrome of inappropriate anti-diuretic hormone (SIADH), is commonly associated with paraneoplastic syndrome in malignancy, especially involving the lung and gastrointestinal system [1]. However, SIADH associated with thymic carcinoma is rare [2, 3]. There has been a previous report highlighting SIADH associated with thymic carcinoma associated with brain metastasis [4]. However, SIADH associated with primary thymic carcinoma alone is poorly documented [5, 6].

We would like to report a case of a patient with symptomatic SIADH as the early presentation of poorly differentiated thymic carcinoma, then shows complete resolution after undergoing surgical intervention and completing the course of adjuvant ‘paclitaxel-carboplatin’ therapy.

## Case report

**Figure 1:**
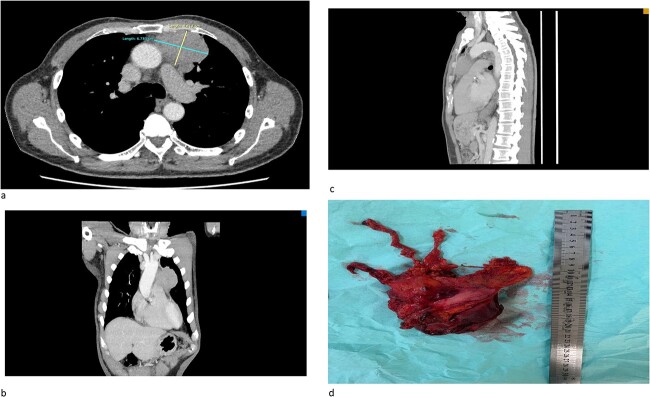
(a) An axial cut of a contrast-enhanced computed tomography (CECT) scan thorax showing an ill-defined anterior mediastinal mass measuring 4.8 cm × 6.7 cm. (b) A coronal view of a CT scan showing an ill-defined anterior mediastinal mass infiltrating the left upper and middle lobe lung. (c) A sagittal view of a CT scan showing an ill-defined anterior mediastinal mass. (d) A successful total thymectomy with R0 resection (microscopically margin-free resection) through a primary median sternotomy.

We would like to report a case of a 67-year-old male with no known co-morbidity who initially presented with generalized fatigue and hemoptysis. The serial bloods results are consistent with persistent hyponatremia. The patient was diagnosed with a duodenal ulcer after orogastroduodenoscopy and SIADH in view of persistent hyponatremia. Initial chest X-ray showed incidental findings of a widened mediastinum, and computed tomography (CT) scan of the thorax confirmed the presence of anterior mediastinal mass (Fig. 1a-c). CT-guided biopsy was inconclusive of the diagnosis and hence referred for surgical intervention. Serum osmolarity, urine osmolarity, and urine sodium were normal despite being followed up with SIADH. CT brain showed no evidence of intracranial involvement.

The patient successfully underwent total thymectomy with pericardium, lower part of upper lobe, and upper part of the lower lobe of the left lung en bloc resection (Fig. 1d). The patient was discharged well. The histopathology (HPE) specimens were reported as poorly differentiated cancer with lung invasion (T3N1MX). He was then referred to the oncology team, and successfully completed four cycles of adjuvant chemotherapy using the ‘paclitaxel carboplatin regime’. His follow-up investigations showed complete resolution of hyponatremia and his symptoms revolved after completion of chemotherapy.

## Discussion

Thymic epithelial tumours (TET), are rare anterior mediastinal tumours, raising from the thymus. It is divided into benign and malignant tumours, of which, thymoma, a benign tumour, is the commonest form of TET. However, thymic carcinoma is rare [2, 3]. United States (US) cancer registry data show that the overall incidence of thymoma is 0.13 per 100 000 person/year [7]. However, the development of hyponatremia as a presentation of SIADH in primary thymic carcinoma is poorly documented in clinical practice, as hyponatremia is usually presented as a paraneoplastic syndrome in thymic carcinoma with involvement of distant metastasis to the brain and lung [3, 4]. The management of para-neoplastic SIADH includes a total resection of the tumour removal and adjuvant chemotherapy [8]. As in this case, the patient presented early with symptomatic hyponatremia secondary to SIADH due to poorly differentiated thymic carcinoma, and a follow-up resolution of hyponatremia after total RO (microscopically margin-free resection) of the tumour and adjuvant chemotherapy.

## Conclusion

Hyponatremia or SIADH can be presented as part of a paraneoplastic syndrome in a poorly differentiated thymic carcinoma, however, it is very rare. A complete resolution of hyponatremia can be achieved after complete removal of the tumour and adjuvant chemotherapy.

## Data Availability

No additional data are available.
